# Association of Potassium Level at Discharge with Long-Term Mortality in Hospitalized Patients with Heart Failure

**DOI:** 10.3390/jcm11247358

**Published:** 2022-12-11

**Authors:** Yusuke Miura, Satoshi Higuchi, Takashi Kohno, Yasuyuki Shiraishi, Mitsunobu Kitamura, Yuji Nagatomo, Makoto Takei, Shintaro Nakano, Ayumi Goda, Kyoko Soejima, Shun Kohsaka, Tsutomu Yoshikawa

**Affiliations:** 1 Department of Cardiovascular Medicine, Kyorin University Hospital, Tokyo 181-8611, Japan; 2 Division of Cardiology, Department of Medicine, Showa University School of Medicine, Tokyo 142-8555, Japan; 3 Department of Cardiology, Keio University School of Medicine, Tokyo 160-8582, Japan; 4 Department of Cardiology, Sakakibara Heart Institute, Tokyo 183-0003, Japan; 5 Department of Cardiology, National Defense Medical College, Tokorozawa 359-8513, Japan; 6 Department of Cardiology, Tokyo Saiseikai Central Hospital, Tokyo 108-0073, Japan; 7 Department of Cardiology, Saitama Medical University, International Medical Center, Saitama 350-0495, Japan

**Keywords:** heart failure, serum potassium, dyskalemia, cardiac death, sudden death

## Abstract

Dyskalemia (hypokalemia and hyperkalemia) is a common comorbidity of heart failure (HF). Although dyskalemia is associated with poor prognosis, different prognostic impacts of hypo- and hyperkalemia remain vastly unclear. This study investigated the association of dyskalemia with prognosis in HF patients, especially the mode of death and left ventricular ejection fraction (LVEF). The multicenter study included 3398 patients hospitalized for HF. Patients were divided into three groups based on serum potassium levels at discharge: hypokalemia (<3.5 mEq/L; *n* = 115 (3.4%)), normokalemia (3.5–5.0 mEq/L; *n* = 2960 (87.1%)), and hyperkalemia (≥5.0 mEq/L; *n* = 323 (9.5%)). Two-year all-cause, cardiac, and non-cardiac mortality was evaluated. Association of serum potassium with two-year mortality demonstrated a U-shaped curve, with a worse prognosis for patients with hypokalemia. All-cause mortality at two-years did not differ among the three groups. Hypokalemia was associated with 2-year cardiac death (adjusted hazard ratio (HR), 2.60; 95% confidence interval (CI), 1.20–5.64) in HF with reduced ejection fraction (HFrEF; LVEF < 40%), but not in non-HFrEF. Regardless of LVEF, hyperkalemia was not independently associated with any mortality. Hypokalemia was independently associated with cardiac death, particularly in HFrEF patients. Such an association was not observed in hyperkalemia regardless of LVEF.

## 1. Introduction

Dyskalemia, namely, hypokalemia or hyperkalemia, is one of common comorbidities in patients with heart failure (HF) [1,2]. Hypokalemia can be exacerbated by diuretics use [3], leading to lethal ventricular arrhythmias [4], whereas hyperkalemia often leads to withholding prescriptions or up-titration of optimal medical therapy, such as mineralocorticoid receptor antagonists (MRA) and renin–angiotensin system inhibitors (RAS-I), both of which can induce hyperkalemia [5]. While both the European and United States clinical practice guidelines recommend closely monitoring serum potassium levels, they also emphasize the evidence–practice gap in appropriate management and the effects on outcomes of dyselectrolytemia in patients with HF [6,7]. The relationship between serum potassium levels and clinical adverse events is reportedly U-shaped, and both low and high potassium levels are associated with mortality and morbidity in HF patients [8,9,10]. However, it has been unknown whether dyskalemia contributes to poor prognosis directly or indirectly through other factors, such as comorbidities or through the administration of optimal medical therapy and diuretics [11]. This retrospective cohort study aimed to investigate the association of hypo- and hyperkalemia with prognosis in HF patients, concentrating on mode of death and left ventricular ejection fraction (LVEF).

## 2. Materials and Methods

### 2.1. Study Population and Protocol

Data from the West Tokyo Heart Failure (WET-HF) registry were retrospectively analyzed. The WET-HF design was previously reported [12]. Briefly, the WET-HF is a prospective multicenter registry designed to collect data regarding clinical characteristics and outcomes of patients hospitalized for acute HF (AHF). AHF was diagnosed by cardiologists at each institution based on the Framingham criteria [13], and 4000 consecutive patients with AHF across 6 academic hospitals were registered between 2006 and 2017. Patients who died during index hospitalization or had undergone hemodialysis were excluded. Individuals without recorded serum potassium levels at discharge and follow-up information were also excluded.

The current study protocol conforms to the 1975 Declaration of Helsinki and is in line with the Ethical Guidelines for Epidemiological Research established by the Japanese government. This study was approved by the ethics committee at each institution and was registered on the University Medical Information Network (UMIN 000001171). Written or oral informed consent was obtained from each patient before registration.

### 2.2. Data Collection

Data on patient characteristics and outcomes, including all-cause, cardiac, and non-cardiac mortalities, were collected by medical doctors and well-trained clinical researchers. LVEF was assessed using Simpson’s biplane method during the index hospitalization after stabilization of HF symptoms. Information on oral medication for HF such as diuretics, beta-blockers, RAS-I, and MRA was recorded at the time of discharge. The formula used to convert other loop diuretics to furosemide equivalents was as follows: furosemide 20 mg = azosemide 30 mg = torsemide 4 mg [14,15]. High-dose loop diuretic intake was defined as a daily dose of >40 mg furosemide equivalent [16]. During the study period, angiotensin receptor–neprilysin inhibitors (ARNI) and sodium-glucose cotransporter 2 inhibitors were not approved in Japan. Data were entered into an electronic data capturing system, which confirms a robust data query engine and system validations for data quality.

### 2.3. Variable Definitions

Hypokalemia, normokalemia, and hyperkalemia were defined as serum potassium levels of <3.5, 3.5–5.0, and ≥5.0 mEq/L, respectively, according to the cutoff values used in previous studies [8,10,17]. HF with reduced ejection fraction (HFrEF) was defined as LVEF of less than forty percent. Combination medical therapy was defined as the co-prescription of RAS-I and beta-blockers. MRA was not included in the combination medical therapy as MRA prescription rate was relatively low (22–29%) in the majority of HF registries [18,19,20]. Cachexia was defined as a combination of body mass index <20 kg/m^2^ and at least one of the following biochemical abnormalities: C-reactive protein >5 mg/L, hemoglobin <120 g/L, and/or albumin <32 g/L, according to a previous study [21].

### 2.4. Study Endpoints

The study endpoints included 2-year all-cause, cardiac, and non-ca deaths. All deaths were reviewed and subsequently categorized into cardiac or non-cardiac deaths, both of which were determined by central committee members based on the 2014 American College of Cardiology or American Heart Association key data elements and definitions for cardiovascular endpoint events in clinical trials [22]. In addition, we analyzed sudden cardiac death (SCD), considering the relationship between dyskalemia and lethal ventricular arrhythmias. SCD was defined as unexpected or unexplained death in a previously stable patient or death from documented or presumed cardiac arrhythmia without a clear non-cardiovascular cause, including death of a comatose patient after attempted resuscitation [22,23].

### 2.5. Statistical Analysis

Categorical variables were expressed as counts and percentages. Continuous values that followed a normal distribution are presented as mean ± standard deviation, whereas non-normally distributed data variables are presented as median and interquartile range. Baseline characteristics among the three groups were compared using analysis of variance or the Kruskal–Wallis test for continuous variables, and Fisher’s exact or the Chi-squared (χ^2^) test for categorical variables, as appropriate. Kaplan–Meier curves with the log-rank test were performed to compare the association between the combination medical therapy and prognosis of each potassium group. Univariate and multivariate Cox regression analyses were used to evaluate the risk of each endpoint and expressed as a hazard ratios (HR) with a 95% confidence interval (CI). The risks of hypo- and hyperkalemia were compared with that of normokalemia because of the U-shaped-curve association between serum potassium levels and 2-year all-cause mortality. Additional HFrEF-stratified analyses were performed. Statistical significance was set at a *p*-value of <0.05. All statistical analyses were performed using EZR (Easy R; Jichi Medical University, Saitama Medical Center, Japan) [24] which is a graphical user interface for R (The R Foundation for Statistical Computing) and Stata software, version 14 (Stata Corp; College Station, TX, USA).

## 3. Results

### 3.1. Patients Characteristics

Of 4000 patients enrolled in the WET-HF registry, 295 who died during the index hospitalization or had undergone hemodialysis and 307 without recorded serum potassium levels at discharge and follow-up information were excluded. Finally, this study included 3398 patients (age, 74 ± 13 years; females, 40.0%; HFrEF, 39.6%). Table 1 shows patient characteristics. The mean potassium level at discharge was 4.3 ± 0.5 mEq/L. Hypokalemia, normokalemia, and hyperkalemia were observed in 115 (3.4%), 2960 (87.1%), and 323 (9.5%) patients, respectively. Hypokalemia was observed more frequently in older women and patients who had been previously hospitalized for HF. Patients with hypokalemia had higher estimated glomerular filtration rates and systolic blood pressure; however, the prescription rate of the combination medical therapy was lower and the prevalence of New York Heart Association class ≥III was higher. Furthermore, the prescription rates of MRA and thiazide diuretics and the prevalence of cachexia were higher in the hypokalemia group.

### 3.2. Association of Potassium with 2-Year Mortality

**Figure 1:**
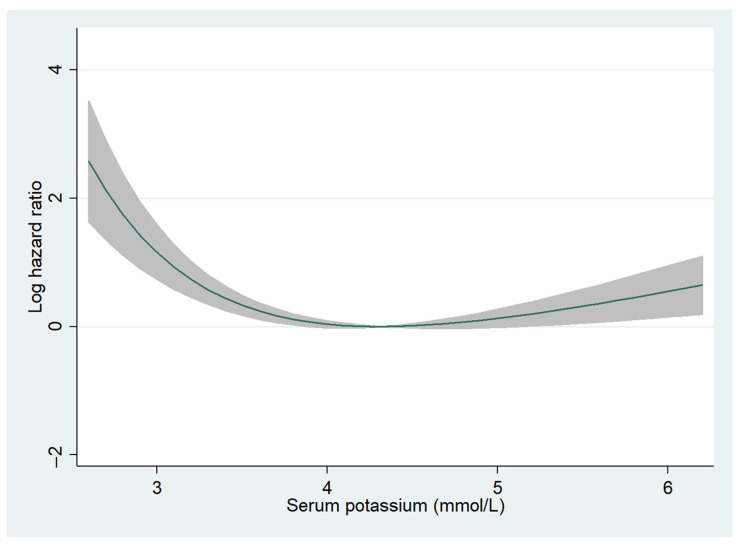
Fractional polynomials for serum potassium levels and 2-year all-cause mortality. The relationship between serum potassium levels and 2-year all-cause mortality is shown. The Y-axis indicates log hazard ratio. The shaded areas represent 95% confidence intervals.

The median follow-up period was 726 (288–1131) days, and 2-year all-cause mortality was observed in 543 (16.0%) patients (hypokalemia, *n* = 28 (24.3%); normokalemia, *n* = 461 (15.6%); and hyperkalemia, *n* = 54 (16.7%)). Fractional polynomials demonstrated a U-shaped curve, with higher 2-year all-cause mortality especially for patients with hypokalemia (Figure 1). In the univariate Cox regression analysis, hypokalemia was significantly associated with higher 2-year all-cause mortality (HR, 1.90; 95% CI, 1.29–2.78; *p* = 0.001) but not in the multivariate Cox regression analysis (HR, 1.34; 95% CI, 0.83–2.17; *p* = 0.228) (Table S1). Additionally, in patients with HFrEF, hypokalemia was significantly correlated with a higher 2-year all-cause mortality in univariate (HR, 2.49; 95% CI, 1.36–4.58; *p* = 0.003) but not in multivariate (HR, 1.70; 95% CI, 0.81–3.59; *p* = 0.160) Cox regression analyses (Table S2). In patients without HFrEF, hypokalemia was not associated with all-cause mortality (Table S3). Hyperkalemia was not associated with all-cause mortality regardless of LVEF (Tables S1–S3).

**Figure 2:**
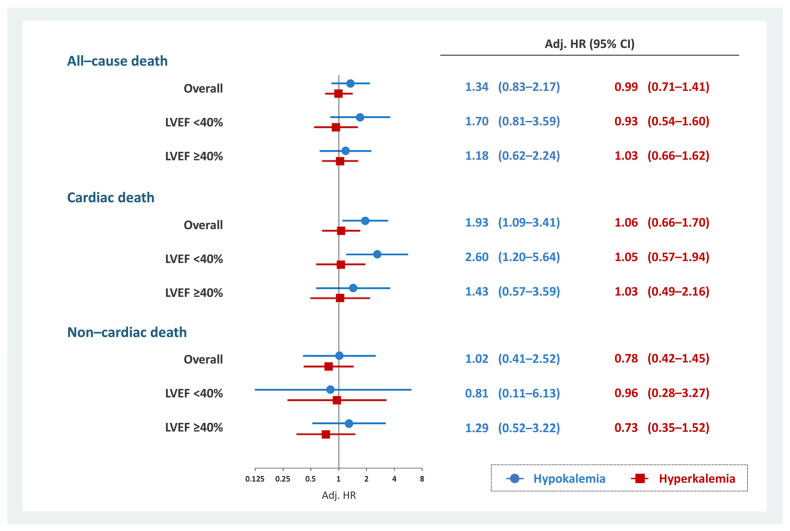
Multivariate Cox regression analysis for each prognosis and dyskalemia. Hypokalemia was significantly associated with 2-year cardiac death in patients with HFrEF but not in those with non-cardiac death. Hyperkalemia was not associated with prognosis. Models were adjusted for age, COPD, ischemic heart disease, eGFR at discharge, combination medical therapy, high-dose loop diuretics, thiazide diuretics, New York Heart Association functional class III/IV, and cachexia. CI, confidence interval; COPD, chronic obstructive pulmonary disease; eGFR, estimated glomerular filtration rate; HFrEF, heart failure with reduced ejection fraction; HR, hazard ratio; LVEF, left ventricular ejection fraction.

Cardiac mortality at 2-years was observed in 278 (8.2%) patients (hypokalemia, *n* = 15 (13.0%); normokalemia, *n* = 233 (7.9%); and hyperkalemia, *n* = 30 (9.3%)). In the overall cohort, hypokalemia was associated with 2-year cardiac death (HR, 1.93; 95% CI, 1.09–3.41; *p* = 0.025) (Table S4). This association persisted in patients with HFrEF (HR, 2.60; 95% CI, 1.20–5.64; *p* = 0.015) but not in patients without HFrEF (HR, 1.43; 95% CI, 0.57–3.59; *p* = 0.443) (Tables S5 and S6). Hyperkalemia was not associated with cardiac death regardless of LVEF (Tables S4–S6). In addition, no significant association existed between non-cardiac death and hypokalemia or hyperkalemia (Tables S7–S9). The results of the multivariate Cox regression analysis evaluating the association between dyskalemia and all-cause, cardiac, and non-cardiac deaths is summarized in Figure 2.

SCD was observed in 95 (2.8%) patients from the overall cohort (hypokalemia, *n* = 7 (6.1%); normokalemia, *n* = 78 (2.6%); and hyperkalemia, *n* = 10 (3.1%)). Univariate Cox regression analysis indicated that hypokalemia tended to be associated with SCD in the overall cohort (HR, 2.02; 95% CI, 0.93–4.38; *p* = 0.075), and this association was significant in patients with HFrEF (HR, 3.96; 95% CI, 1.56–10.09; *p* = 0.004). Hyperkalemia was not associated with SCD regardless of LVEF (Figure S1).

### 3.3. Prescription Rate and Impact of Combination Medical Therapy

**Figure 3:**
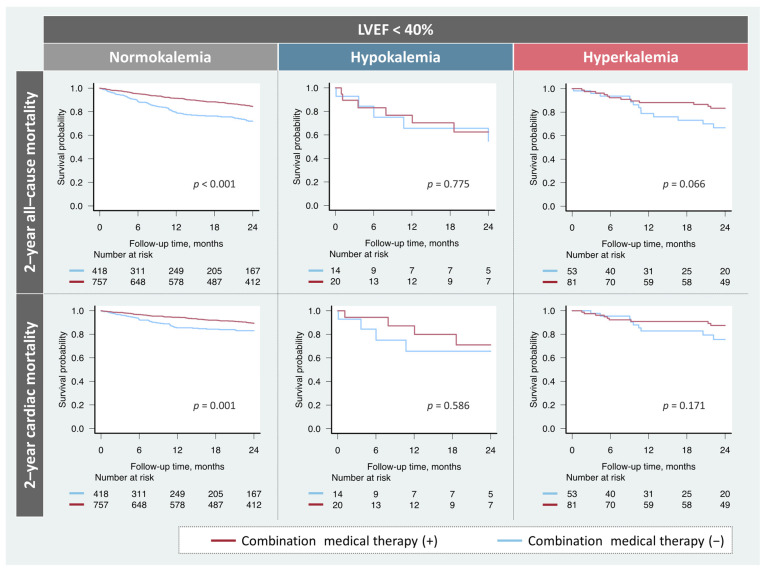
Impact of the combination medical therapy on prognosis based on potassium levels. Combination medical therapy was significantly associated with a favorable prognosis in patients with normokalemia. However, these findings were not observed in patients with hypokalemia. LVEF, left ventricular ejection fraction.

Among the patients with HFrEF, the prescription rates of the combination therapy did not significantly differ (hypokalemia, *n* = 20 (59%); normokalemia, *n* = 757 (64%); hyperkalemia, *n* = 81 (60%); *p* = 0.545). The prognostic impact of the combination medical therapy based on potassium levels in patients with HFrEF is shown in Figure 3. Treatment was significantly associated with favorable prognosis in patients with HFrEF and normokalemia. While patients with HFrEF and hyperkalemia tended to be correlated with better prognosis, no association of the combination therapy with any endpoints was observed in patients with HFrEF and hypokalemia.

## 4. Discussion

In one of the largest Japanese multicenter registry studies, we demonstrated that hypokalemia was associated with an older age, the female sex, and frequent use of thiazide diuretics and less frequent use of the combination medical therapy. Hypokalemia in HFrEF was independently associated with higher cardiac mortalities, whereas hypokalemia in non-HFrEF and hyperkalemia in both HFrEF and non-HFrEF were not associated with these endpoints. To the best of our knowledge, this is the first study to report the different prognostic impact of hypo- and hyperkalemia in patients with HF.

### 4.1. Differential Impact of Hypokalemia and Hyperkalemia on Prognosis

Consistent with previous studies [8,9,10,25,26,27], hypokalemia (K^+^ < 3.5 mEq/L) was associated with a poor prognosis in the present study. Even after adjustment for several poor prognosis-related covariates such as older age, cachexia, high-dose loop diuretics, and frequent use of thiazide diuretic, hypokalemia was independently associated with higher cardiac mortality, but not non-cardiac mortality in patients with HFrEF. This association was not observed in patients with non-HFrEF. Our findings suggested that the mode of death and LVEF should be considered when discussing the prognostic impact of hypokalemia in patients with HF. In this study, the combination medical therapy was not associated with a favorable prognosis in patients with HFrEF and hypokalemia. These results might be due to beta error or suggested that a certain number of preventable deaths in individuals with hypokalemia might not be affected by the combination medical therapy. Unlike ARNI, RAS-I could not prevent SCD [28], which was more frequently observed in patients with HFrEF and hypokalemia.

In addition to the prognostic impact of hypokalemia, several observational studies have reported that hyperkalemia was associated with poor prognosis in patients with HF, describing a U-shaped relationship between dyskalemia and prognosis [8,9,10]. However, whether these associations are direct or indirect remains unclear. Hyperkalemia has been reported to be an independent risk factor for dose reduction of RAS-I [29,30]. Rossignol et al. [31] reported that both hypo- and hyperkalemia were associated with higher mortality; however, after adjustment for discontinuation of RAS-I or MRA, hypokalemia was associated with mortality whereas hyperkalemia was not, which was concordant with our study findings. According to the fractional polynomials, hyperkalemia seemed to be a negative prognostic factor; however, its crude association with mortality was not significant. This may be explained by the equivalent RAS-I prescription rates between the hyperkalemia and normokalemia groups. Therefore, hyperkalemia may cause a reduction or discontinuation of RAS-I and/or MRA, reducing the potential efficacy of these agents, indirectly resulting in the poor prognosis of patients with HF. Whether the use of RAS-I and/or MRA can improve the prognosis of patients with HFrEF and hyperkalemia remains unclear; this should be prioritized in future studies.

The different prognostic significance of hypo- and hyperkalemia may also be explained from the viewpoint of life-threatening arrhythmias. Hypokalemia leads to prolonged action potential duration (APD), reduced repolarization reserve, and early afterdepolarizations, which can induce ventricular fibrillation leading to SCD via torsades de pointes or polymorphic ventricular tachycardia [32]. In contrast, hyperkalemia enhances the repolarization reserve by inducing APD shortening and post-repolarization refractoriness. Even moderate hypokalemia (2.5–3.0 mEq/L) is highly arrhythmogenic, whereas serious electrocardiographic changes (P-wave disappearance or QRS widening) are infrequent in hyperkalemia, unless very severe ([K^+^] > 7.0 mEq/L) [33].

### 4.2. Patient Characteristics and Therapeutic Targets of Hypokalemia

Hypokalemia is associated with prognostic and modifiable factors in HF. The dyselectrolytemia was related to diuretic resistance [3], frequent use of diuretics, especially thiazide diuretics [34], and less frequent use of RAS-I [35], which was consistent with the findings in our contemporary hospitalized HF cohort. Although the prescription rate of MRA was higher in patients with hypokalemia than in those with hyperkalemia, it was still overall lower compared to that of other studies from Western countries [36]. Additionally, the present study demonstrated that cachexia was independently associated with hypokalemia, which has not been evaluated in previous HF cohorts. Elucidating whether potassium supplements and/or interventions targeted at these modifiable factors, such as thiazide diuretic discontinuation, potassium-sparing diuretic initiation, and dietary intervention, could improve the prognosis of patients with HF and hypokalemia should be important in future studies.

### 4.3. Limitations

Our study had some limitations. First, this was a retrospective observational study; thus, causal relationships could not be proven and residual confounding factors may have existed. In addition, we could not identify whether sudden deaths were caused by lethal arrhythmias nor could we precisely evaluate the association between lethal arrhythmias and dyskalemia. Second, this study was based on a single serum potassium measurement at discharge, without any follow-up data on potential changes over time. Additionally, data on the prescription of potassium supplements or potassium binders were not collected. Serum potassium values can fluctuate depending on diuretic dose adjustments, potassium intake, and potassium binder use. In a recent study on the long-term potassium dynamics in patients with HF, dyskalemia persistence was associated with higher mortality compared to normalization from dyskalemia [8]. In future research, the prognostic impact of sequential potassium measurements of patients with HF after discharge must be investigated. Third, our findings might be unique to Japan, where the prevalence of ischemic cardiomyopathy and the rate of ICD or CRT impanation are low compared to Western countries [37]. Fourth, the statistical power may not have been sufficient to detect any negative outcomes among patients with hypokalemia.

## 5. Conclusions

Hypokalemia was associated with cardiac death, especially in patients with HFrEF, whereas hyperkalemia was not. Hypokalemia and hyperkalemia demonstrated different clinical impacts on the prognosis of patients with HF. Although maintenance of the normal potassium range should be a therapeutic goal for patients with HF and hypokalemia, future studies to establish management strategies to improve prognosis are necessary.

## Figures and Tables

**Table 1:** Patient Characteristics.

	All (*n* = 3398)	Hypokalemia (*n* = 115)	Normokalemia (*n* = 2960)	Hyperkalemia (*n* = 323)	*p* Value
Age, years	74 ± 13	77 ± 13	74 ± 13	75 ± 12	0.015
Female, *n* (%)	1360 (40)	58 (50)	1191 (40)	111 (34)	0.008
Hypertension, *n* (%)	2225 (65)	76 (66)	1928 (65)	221 (68)	0.501
Dyslipidemia, *n* (%)	1294 (38)	41 (36)	1127 (38)	126 (39)	0.819
Diabetes Mellitus, *n* (%)	1128 (33)	33 (29)	969 (33)	126 (39)	0.047
History of HF, *n* (%)	999 (29)	54 (47)	852 (29)	93 (29)	<0.001
COPD, *n* (%)	163 (5)	4 (3)	141 (5)	18 (6)	0.716
Stroke, *n* (%)	446 (13)	18 (16)	386 (13)	42 (13)	0.656
LVEF, %	45 ± 15	47 ± 14	45 ± 15	44 ± 15	0.174
LVEF <40%, *n* (%)	1344 (40)	34 (30)	1175 (40)	134 (41)	0.059
Etiologies of heart failure					
IHD, *n* (%)	971 (29)	26 (23)	843 (28)	102 (32)	0.183
DCM, *n* (%)	486 (14)	7 (6)	430 (15)	49 (15)	0.025
VHD, *n* (%)	889 (26)	39 (34)	778 (26)	72 (22)	0.049
Others, *n* (%)	1052 (31)	43 (37)	909 (31)	100 (31)	0.314
AF/AFL, *n* (%)	1706 (50)	62 (54)	1501 (51)	143 (44)	0.189
Pacemaker, *n* (%)	264 (8)	13 (11)	231 (8)	20 (6)	0.217
ICD, *n* (%)	124 (4)	8 (7)	103 (3)	13 (4)	0.137
CRT, *n* (%)	58 (2)	4 (3)	47 (2)	7 (2)	0.166
Status at discharge					
Body mass index, kg/m^2^	22 ± 4	21 ± 4	22 ± 4	21 ± 4	0.005
Systolic blood pressure, mmHg	112 ± 17	116 ± 19	112 ± 17	111 ± 18	0.019
Diastolic blood pressure, mmHg	62 ± 11	63 ± 13	61 ± 11	62 ± 12	0.345
Heart rate, bpm	71 ± 13	73 ± 14	71 ± 12	71 ± 13	0.122
NYHA III/IV, *n* (%)	701 (21)	38 (33)	589 (20)	74 (23)	0.002
Cachexia, *n* (%)	759 (22)	38 (33)	645 (22)	76 (24)	0.012
Laboratory data at discharge					
Hemoglobin, g/dL	12.2 ± 2.2	11.5 ± 2.0	12.2 ± 2.1	12.2 ± 2.4	0.003
eGFR, mL/min/1.73 m^2^	51.0 (36.2–64.6)	55.4 (35.9–67.1)	51.8 (37.4–65.5)	40.5 (28.8–55.0)	<0.001
Albumin, g/dL	3.5 ± 0.5	3.3 ± 0.5	3.5 ± 0.5	3.5 ± 0.5	<0.001
Sodium, mEq/L	139 ± 4	140 ± 4	139 ± 3	137 ± 4	<0.001
Potassium, mEq/L	4.3 ± 0.5	3.2 ± 0.2	4.3 ± 0.4	5.3 ± 0.4	<0.001
Medication at discharge					
Combination medical therapy, *n* (%)	1740 (51)	46 (40)	1522 (51)	172 (53)	0.041
RAS-I, *n* (%)	2170 (64)	63 (55)	1891 (64)	216 (67)	0.069
Beta-blockers, *n* (%)	2609 (77)	78 (68)	2275 (77)	256 (79)	0.042
MRA, *n* (%)	1213 (36)	42 (37)	1076 (36)	95 (29)	0.042
Loop diuretics, *n* (%)	2621 (77)	90 (78)	2296 (78)	235 (73)	0.136
Furosemide equivalent dose, mg	20 (20–40)	40 (20–40)	20 (20–40)	20 (20–40)	<0.001
High-dose loop diuretics, *n* (%)	276 (8)	19 (17)	239 (8)	18 (6)	0.004
Thiazide diuretics, *n* (%)	244 (7)	18 (16)	210 (7)	16 (5)	0.002

HF, heart failure; COPD, chronic obstructive pulmonary disease; LVEF, left ventricular ejection fraction; IHD, ischemic heart disease; DCM, dilated cardiomyopathy; VHD, valvular heart diseases; AF, atrial fibrillation; AFL, atrial flutter; ICD, implantable cardioverter defibrillator; CRT, cardiac resynchronization therapy; NYHA, New York Heart Association; eGFR, estimated glomerular filtration rate; RAS-I, renin–angiotensin system inhibitors; MRA, mineralocorticoid-receptor antagonists.

## Data Availability

The data presented in this study are available on request from the corresponding author.

## Supplementary Materials

The following supporting information can be downloaded at: https://www.mdpi.com/article/10.3390/jcm11247358/s1, Table S1: Cox regression analysis for 2-year all-cause mortality (overall); Table S2: Cox regression analysis for 2-year all-cause mortality (LVEF < 40%); Table S3: Cox regression analysis for 2-year all-cause mortality (LVEF ≥ 40%); Table S4: Cox regression analysis for 2-year cardiac mortality (overall); Table S5: Cox regression analysis for 2-year cardiac mortality (LVEF < 40%); Table S6: Cox regression analysis for 2-year cardiac mortality (LVEF ≥ 40%); Table S7: Cox regression analysis for 2-year non-cardiac mortality (overall); Table S8: Cox regression analysis for 2-year non-cardiac mortality (LVEF < 40%); Table S9: Cox regression analysis for 2-year non-cardiac mortality (LVEF ≥ 40%); Figure S1: Association between sudden cardiac death and dyskalemia.
