# Supplementary material for: Association of Potassium Level at Discharge with Long-Term Mortality in Hospitalized Patients with Heart Failure
Source: J Clin Med. 2022 Dec 11;11(24):7358. doi: 10.3390/jcm11247358 (PMC9782550; doi:10.3390/jcm11247358)
Supplement: Supplementary file 1 [file jcm-11-07358-s001.zip › jcm-2046267-supplementary.pdf]

Supplementary Materials

Table S1. Cox regression analysis for 2–year all–cause mortality (overall)

	Univariate			Multivariate		
	HR	95%CI	p value	HR	95%CI	p value
Age	1.05	1.04–1.06	<0.001	1.02	1.01–1.03	<0.001
Female	1.04	0.87–1.23	0.696			
Hypertension	0.92	0.77–1.09	0.321			
Dyslipidemia	0.91	0.77–1.09	0.311			
Diabetes Mellitus	1.10	0.92–1.31	0.313			
COPD	1.90	1.41–2.57	<0.001	1.78	1.24–2.55	0.002
IHD	1.43	1.20–1.71	<0.001	1.25	1.01–1.55	0.039
NYHA III/IV	2.59	2.18–3.08	<0.001	1.76	1.42–2.18	<0.001
Cachexia	2.49	2.08–2.98	<0.001	1.90	1.53–2.36	<0.001
Hemoglobin level at discharge (an increase of 1.0 g/dL)	0.77	0.73–0.80	<0.001			
eGFR level at discharge (an increase of 1.0 mL/min/1.73m^2)	0.98	0.98–0.99	<0.001	0.99	0.98–0.99	<0.001
LVEF (an increase of 1%)	0.99	0.99–1.00	0.136			
Combination medical therapy	0.63	0.53–0.74	<0.001	0.83	0.68–1.03	0.087
MRA	0.95	0.80–1.13	0.582			
High–dose loop diuretics	1.72	1.33–2.21	<0.001	1.39	1.06–1.83	0.019
Thiazide diuretics	2.58	2.02–3.29	<0.001	1.78	1.34–2.36	<0.001
Hypokalemia (reference: normokalemia)	1.90	1.29–2.78	0.001	1.34	0.83–2.17	0.228
Hyperkalemia (reference: normokalemia)	1.03	0.78–1.37	0.832	0.99	0.71–1.41	0.992

COPD, chronic obstructive pulmonary disease; IHD, ischemic heart disease; NYHA, New York Heart Association; eGFR, estimated glomerular filtration rate; LVEF, left ventricular ejection fraction; MRA, mineralocorticoid–receptor antagonists

Table S2. Cox regression analysis for 2–year all–cause mortality (LVEF <40%)

	Univariate			Multivariate		
	HR	95%CI	p value	HR	95%CI	p value
Age	1.06	1.04–1.07	<0.001	1.03	1.01–1.04	<0.001
Female	1.22	0.92–1.61	0.696			
Hypertension	0.92	0.70–1.19	0.514			
Dyslipidemia	1.08	0.83–1.41	0.574			
Diabetes Mellitus	1.31	1.00–1.70	0.048			
COPD	1.45	0.83–2.54	0.193	1.68	0.91–3.09	0.098
IHD	1.84	1.42–2.39	<0.001	1.26	0.91–1.73	0.158
NYHA III/IV	2.70	2.06–3.54	<0.001	1.89	1.36–2.61	<0.001
Cachexia	3.04	2.30–4.02	<0.001	1.86	1.33–2.61	<0.001
Hemoglobin level at discharge (an increase of 1.0 g/dL)	0.72	0.67–0.76	<0.001			
eGFR level at discharge (an increase of 1.0 mL/min/1.73m^2)	0.98	0.97–0.98	<0.001	0.99	0.98–1.00	0.115
Combination medical therapy	0.49	0.38–0.64	<0.001	0.78	0.56–1.08	0.133
MRA	0.82	0.63–1.07	0.141			
High–dose loop diuretics	1.54	1.04–2.27	0.031	1.44	0.96–2.17	0.081
Thiazide diuretics	3.22	2.25–4.61	<0.001	2.17	1.45–3.27	<0.001
Hypokalemia (reference: normokalemia)	2.49	1.36–4.58	0.003	1.70	0.81–3.59	0.160
Hyperkalemia (reference: normokalemia)	1.15	0.76–1.74	0.515	0.93	0.54–1.60	0.792

LVEF, left ventricular ejection fraction; COPD, chronic obstructive pulmonary disease; IHD, ischemic heart disease; NYHA, New York Heart Association; eGFR, estimated glomerular filtration rate; MRA, mineralocorticoid–receptor antagonists

Table S3. Cox regression analysis for 2–year all–cause mortality (LVEF ≥40%)

	Univariate			Multivariate		
	HR	95%CI	p value	HR	95%CI	p value
Age	1.05	1.04–1.07	<0.001	1.02	1.00–1.03	0.027
Female	0.97	0.78–1.22	0.820			
Hypertension	0.93	0.74–1.18	0.557			
Dyslipidemia	0.80	0.64–1.02	0.068			
Diabetes Mellitus	0.95	0.75–1.21	0.701			
COPD	2.14	1.48–3.08	<0.001	1.83	1.16–2.89	0.010
IHD	1.16	0.90–1.49	0.257	1.10	0.81–1.49	0.558
NYHA III/IV	2.51	2.00–3.15	<0.001	1.62	1.21–2.15	<0.001
Cachexia	2.22	1.75–2.81	<0.001	1.89	1.42–2.51	<0.001
Hemoglobin level at discharge (an increase of 1.0 g/dL)	0.79	0.74–0.84	<0.001			
eGFR level at discharge (an increase of 1.0 mL/min/1.73m^2)	0.98	0.98–0.99	<0.001	0.98	0.98–0.99	<0.001
Combination medical therapy	0.69	0.55–0.87	0.002	0.78	0.59–1.04	0.092
MRA	1.02	0.81–1.30	0.851			
High–dose loop diuretics	1.86	1.33–2.60	<0.001	1.36	0.93–1.97	0.111
Thiazide diuretics	2.19	1.68–3.05	<0.001	1.48	0.99–2.20	0.051
Hypokalemia (reference: normokalemia)	1.65	1.01–2.70	0.045	1.18	0.62–2.24	0.620
Hyperkalemia (reference: normokalemia)	0.95	0.64–1.39	0.774	1.03	0.66–1.62	0.894

LVEF, left ventricular ejection fraction; COPD, chronic obstructive pulmonary disease; IHD, ischemic heart disease; NYHA, New York Heart Association; eGFR, estimated glomerular filtration rate; MRA, mineralocorticoid–receptor antagonists

Table S4. Cox regression analysis for 2–year cardiac mortality (overall)

	Univariate			Multivariate		
	HR	95%CI	p value	HR	95%CI	p value
Age	1.04	1.03–1.05	<0.001	1.01	0.99–1.02	0.374
Female	0.99	0.78–1.27	0.966			
Hypertension	0.79	0.62–1.00	0.054			
Dyslipidemia	0.93	0.73–1.19	0.584			
Diabetes Mellitus	1.17	0.92–1.49	0.209			
COPD	1.50	0.94–2.39	0.088	1.63	0.97–2.72	0.065
IHD	1.74	1.37–2.21	<0.001	1.72	1.29–2.29	<0.001
NYHA III/IV	2.74	2.16–3.49	<0.001	1.98	1.48–2.65	<0.001
Cachexia	2.15	1.67–2.77	<0.001	1.65	1.22–2.24	0.001
Hemoglobin level at discharge (an increase of 1.0 g/dL)	0.81	0.76–0.86	<0.001			
eGFR level at discharge (an increase of 1.0 mL/min/1.73m^2)	0.98	0.98–0.99	<0.001	0.99	0.98–0.99	0.004
LVEF (an increase of 1%)	0.98	0.97–0.99	<0.001			
Combination medical therapy	0.63	0.50–0.80	<0.001	0.79	0.59–1.05	0.106
MRA	0.88	0.68–1.12	0.293			
High–dose loop diuretics	1.73	1.22–2.45	0.002	1.46	1.01–2.11	0.045
Thiazide diuretics	2.72	1.95–3.80	<0.001	2.03	1.40–2.95	<0.001
Hypokalemia (reference: normokalemia)	2.01	1.19–3.38	0.009	1.93	1.09–3.41	0.025
Hyperkalemia (reference: normokalemia)	1.13	0.78–1.66	0.518	1.06	0.66–1.70	0.801

COPD, chronic obstructive pulmonary disease; IHD, ischemic heart disease; NYHA, New York Heart Association; eGFR, estimated glomerular filtration rate; LVEF, left ventricular ejection fraction; MRA, mineralocorticoid–receptor antagonists

Table S5. Cox regression analysis for 2–year cardiac mortality (LVEF <40%)

	Univariate			Multivariate		
	HR	95%CI	p value	HR	95%CI	p value
Age	1.05	1.04–1.07	<0.001	1.02	1.01–1.04	0.014
Female	1.17	0.83–1.66	0.371			
Hypertension	0.80	0.58–1.11	0.177			
Dyslipidemia	1.03	0.74–1.44	0.844			
Diabetes Mellitus	1.30	0.94–1.81	0.113			
COPD	1.94	1.05–3.59	0.034	1.89	0.95–3.77	0.070
IHD	2.14	1.55–2.96	<0.001	1.63	1.11–2.40	0.013
NYHA III/IV	3.13	2.25–4.36	<0.001	1.97	1.33–2.91	<0.001
Cachexia	2.41	1.69–3.44	<0.001	1.51	1.01–2.28	0.047
Hemoglobin level at discharge (an increase of 1.0 g/dL)	0.74	0.68–0.80	<0.001			
eGFR level at discharge (an increase of 1.0 mL/min/1.73m^2)	0.97	0.96–0.98	<0.001	0.99	0.98–0.99	0.044
Combination medical therapy	0.55	0.40–0.76	<0.001	0.84	0.57–1.25	0.393
MRA	0.76	0.55–1.05	0.098			
High–dose loop diuretics	1.76	1.12–2.76	0.014	1.60	0.99–2.57	0.054
Thiazide diuretics	3.45	2.24–5.31	<0.001	2.25	1.39–3.63	<0.001
Hypokalemia (reference: normokalemia)	2.86	1.40–5.86	0.004	2.60	1.20–5.64	0.015
Hyperkalemia (reference: normokalemia)	1.31	0.80–2.15	0.288	1.05	0.57–1.94	0.882

LVEF, left ventricular ejection fraction; COPD, chronic obstructive pulmonary disease; IHD, ischemic heart disease; NYHA, New York Heart Association; eGFR, estimated glomerular filtration rate; MRA, mineralocorticoid–receptor antagonists

Table S6. Cox regression analysis for 2–year cardiac mortality (LVEF ≥40%)

	Univariate			Multivariate		
	HR	95%CI	p value	HR	95%CI	p value
Age	1.04	1.02–1.06	<0.001	1.01	0.98–1.02	0.872
Female	1.04	0.74–1.46	0.834			
Hypertension	0.83	0.58–1.19	0.321			
Dyslipidemia	0.80	0.55–1.14	0.215			
Diabetes Mellitus	0.96	0.67–1.39	0.839			
COPD	1.21	0.59–2.47	0.603	1.23	0.53–2.86	0.624
IHD	1.14	0.77–1.68	0.519	1.33	0.82–2.14	0.247
NYHA III/IV	2.47	1.74–3.50	<0.001	1.77	1.13–2.78	0.013
Cachexia	2.14	1.48–3.08	<0.001	1.77	1.13–2.79	0.013
Hemoglobin level at discharge (an increase of 1.0 g/dL)	0.83	0.76–0.91	<0.001			
eGFR level at discharge (an increase of 1.0 mL/min/1.73m^2)	0.99	0.98–0.99	0.002	0.99	0.98–1.00	0.082
Combination medical therapy	0.52	0.36–0.76	<0.001	0.55	0.34–0.88	0.013
MRA	0.83	0.56–1.22	0.334			
High–dose loop diuretics	1.63	0.94–2.82	0.080	1.26	0.69–2.29	0.445
Thiazide diuretics	2.08	1.23–3.52	0.006	1.66	0.90–3.06	0.104
Hypokalemia (reference: normokalemia)	1.61	0.75–3.46	0.221	1.43	0.57–3.59	0.443
Hyperkalemia (reference: normokalemia)	0.93	0.51–1.68	0.808	1.03	0.49–2.16	0.949

LVEF, left ventricular ejection fraction; COPD, chronic obstructive pulmonary disease; IHD, ischemic heart disease; NYHA, New York Heart Association; eGFR, estimated glomerular filtration rate; MRA, mineralocorticoid–receptor antagonists

Table S7. Cox regression analysis for 2–year non–cardiac mortality (overall)

	Univariate			Multivariate		
	HR	95%CI	p value	HR	95%CI	p value
Age	1.06	1.04–1.08	<0.001	1.03	1.01–1.05	0.002
Female	1.18	0.89–1.57	0.258			
Hypertension	1.03	0.76–1.38	0.870			
Dyslipidemia	0.86	0.64–1.16	0.329			
Diabetes Mellitus	0.95	0.71–1.29	0.761			
COPD	2.38	1.50–3.78	<0.001	1.95	1.10–3.48	0.023
IHD	1.18	0.87–1.60	0.278	0.90	0.62–1.31	0.593
NYHA III/IV	2.35	1.75–3.15	<0.001	1.47	1.03–2.12	0.036
Cachexia	3.01	2.24–4.05	<0.001	2.22	1.56–3.18	<0.001
Hemoglobin level at discharge (an increase of 1.0 g/dL)	0.70	0.65–0.76	<0.001			
eGFR level at discharge (an increase of 1.0 mL/min/1.73m^2)	0.98	0.98–0.99	<0.001	0.98	0.98–0.99	0.001
LVEF (an increase of 1%)	1.02	1.01–1.03	0.002			
Combination medical therapy	0.63	0.48–0.84	0.002	0.87	0.61–1.23	0.424
MRA	1.02	0.77–1.37	0.881			
High–dose loop diuretics	1.61	1.04–2.49	0.031	1.24	0.77–2.01	0.373
Thiazide diuretics	1.95	1.25–3.05	0.003	1.26	0.75–2.13	0.384
Hypokalemia (reference: normokalemia)	2.07	1.13–3.81	0.019	1.02	0.41–2.52	0.962
Hyperkalemia (reference: normokalemia)	0.90	0.55–1.49	0.684	0.78	0.42–1.45	0.428

COPD, chronic obstructive pulmonary disease; IHD, ischemic heart disease; NYHA, New York Heart Association; eGFR, estimated glomerular filtration rate; LVEF, left ventricular ejection fraction; MRA, mineralocorticoid–receptor antagonists

Table S8. Cox regression analysis for 2–year non–cardiac mortality (LVEF <40%)

	Univariate			Multivariate		
	HR	95%CI	p value	HR	95%CI	p value
Age	1.07	1.04–1.10	<0.001	1.04	1.00–1.08	0.040
Female	1.38	0.80–2.38	0.247			
Hypertension	1.31	0.75–2.30	0.340			
Dyslipidemia	1.15	0.68–1.94	0.605			
Diabetes Mellitus	1.38	0.82–2.33	0.225			
COPD	0.86	0.21–3.53	0.834	1.37	0.32–5.81	0.668
IHD	1.59	0.94–2.68	0.081	0.88	0.44–1.75	0.711
NYHA III/IV	2.01	1.14–3.56	0.016	1.54	0.74–3.18	0.244
Cachexia	5.09	2.93–8.83	<0.001	3.38	1.63–7.01	0.001
Hemoglobin level at discharge (an increase of 1.0 g/dL)	0.64	0.56–0.74	<0.001			
eGFR level at discharge (an increase of 1.0 mL/min/1.73m^2)	0.99	0.98–1.01	0.214	1.00	0.98–1.02	0.819
Combination medical therapy	0.42	0.25–0.71	0.001	0.78	0.39–1.57	0.484
MRA	0.88	0.52–1.48	0.622			
High–dose loop diuretics	0.41	0.10–1.68	0.213	0.42	0.10–1.78	0.239
Thiazide diuretics	2.01	0.86–4.70	0.106	1.37	0.47–3.98	0.570
Hypokalemia (reference: normokalemia)	1.77	0.43–7.29	0.427	0.81	0.11–6.13	0.839
Hyperkalemia (reference: normokalemia)	1.07	0.46–2.50	0.873	0.96	0.28–3.27	0.953

LVEF, left ventricular ejection fraction; COPD, chronic obstructive pulmonary disease; IHD, ischemic heart disease; NYHA, New York Heart Association; eGFR, estimated glomerular filtration rate; MRA, mineralocorticoid–receptor antagonists

Table S9. Cox regression analysis for 2–year non–cardiac mortality (LVEF ≥40%)

	Univariate			Multivariate		
	HR	95%CI	p value	HR	95%CI	p value
Age	1.05	1.03–1.07	<0.001	1.02	0.99–1.04	0.098
Female	1.02	0.73–1.43	0.895			
Hypertension	0.89	0.63–1.28	0.534			
Dyslipidemia	0.78	0.54–1.12	0.176			
Diabetes Mellitus	0.84	0.57–1.22	0.350			
COPD	2.74	1.65–4.56	<0.001	2.23	1.18–4.22	0.013
IHD	1.14	0.78–1.68	0.493	0.94	0.59–1.49	0.799
NYHA III/IV	2.39	1.69–3.38	<0.001	1.48	0.97–2.26	0.067
Cachexia	2.32	1.63–3.31	<0.001	1.92	1.26–2.90	0.002
Hemoglobin level at discharge (an increase of 1.0 g/dL)	0.74	0.68–0.82	<0.001			
eGFR level at discharge (an increase of 1.0 mL/min/1.73m^2)	0.98	0.97–0.99	<0.001	0.98	0.97–0.99	<0.001
Combination medical therapy	0.83	0.59–1.17	0.287	0.93	0.62–1.41	0.744
MRA	1.22	0.86–1.74	0.264			
High–dose loop diuretics	2.33	1.46–3.72	<0.001	1.58	0.94–2.67	0.087
Thiazide diuretics	1.90	1.13–3.21	0.016	1.22	0.67–2.22	0.525
Hypokalemia (reference: normokalemia)	2.03	1.03–4.01	0.040	1.29	0.52–3.22	0.583
Hyperkalemia (reference: normokalemia)	0.84	0.45–1.55	0.571	0.73	0.35–1.52	0.397

LVEF, left ventricular ejection fraction; COPD, chronic obstructive pulmonary disease; IHD, ischemic heart disease; NYHA, New York Heart Association; eGFR, estimated glomerular filtration rate; MRA, mineralocorticoid–receptor antagonists

Figure S1. Association between sudden cardiac death and dyskalemia

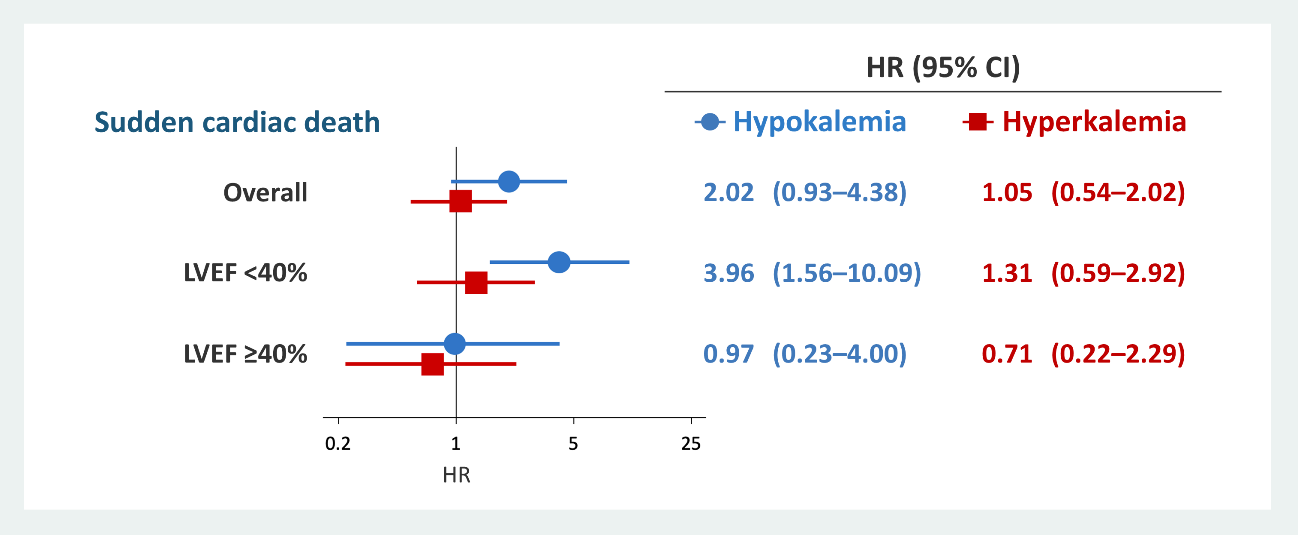

Hypokalemia was significantly associated with sudden cardiac death in patients with HFrEF, while hyperkalemia was not regardless of LVEF.

CI, confidence interval; HR, hazard ratio; LVEF, left ventricular ejection fraction.
